# Terpenyl-Purines from the Sea

**DOI:** 10.3390/md7040833

**Published:** 2009-12-23

**Authors:** Marina Gordaliza

**Affiliations:** Department of Pharmaceutical Chemistry, Pharmacy Faculty, Salamanca University, Campus Miguel de Unamuno, 37007 Salamanca, Spain; E-Mail: mliza@usal.es; Tel.: +34-923-294-528; Fax: +34-923-294-515

**Keywords:** agelasine, agelasimine, ageline, asmarine, alkyl-purine, terpene-purine, terpenylpurine

## Abstract

Agelasines, asmarines and related compounds are natural products with a hybrid terpene-purine structure isolated from numerous genera of sponges (*Agela* sp., *Raspailia* sp.). Some agelasine analogs and related structures have displayed high general toxicity towards protozoa, and have exhibited broad-spectrum antimicrobial activity against a variety of species, including *Mycobacterium tuberculosis*, and also an important cytotoxic activity against several cancer cell lines, including multidrug-resistant ones. Of particular interest in this context are the asmarines (tetrahydro[1,4]diazepino[1,2,3-*g*,*h*]purines), which have shown potent antiproliferative activity against several types of human cancer cell lines. This review summarizes the sources of isolation, chemistry and bioactivity of marine alkylpurines and their bioactive derivatives.

## 1. Introduction

Natural products have been a rich source of agents valued in medicine and are the most productive source of developmental drugs [1–12]. It is widely accepted that more than 80% of drug substances are natural products or have been inspired by a natural compound. Over 100 new products are under clinical development, in particular as anticancer and anti-infective agents [13–15].

Many research groups are dedicated to the isolation and identification of new natural products and other research groups use natural compounds as a models or starting materials for the preparation of derivatives that display some type of beneficial activity for human beings, mainly in the health field [16–20].

The sea is an important source of new natural compounds [21–23]. Some of them have biological, pharmacological or cytotoxic activities [24–27]. Recent research focused on marine natural products has uncovered a useful way to obtain a potentially rich source of drug candidates [28–33], where alkaloids have been found to be more effective in several therapeutic fields. The drug leads based on marine natural products have, however, created unique challenges in scaleable production and structural optimization to evaluate toxicity and enhance biological activity.

Agelasines, asmarines and related compounds are characteristic marine metabolites frequently isolated from sponge genera. Such compounds have attracted the attention of researchers because of their potent biological activities, which have been reported to include antimicrobial [34,35], antiproliferative [36], antileukemic [37], cytotoxic [34,38], antiprotozoal [39,40] and antituberculosis properties [41] and inhibitory effects on the enzymatic reactions of Na^+^,K^+^-ATPase [42]. Another reason that led to the evaluation of genera of sponges is that a significant number of sponge metabolites show promising activities in antifouling assays [43].

This review presents a comprehensive review of the literature published about several aspects of alkylpurine metabolites from marine sponges, including the structure, isolation, biological activity and chemistry of marine metabolites with a terpene-purine structure, as well as their ability to act as antifouling agents. Emphasis is placed on their biological activity and chemistry.

## 2. Alkylpurine Structures

The terpene class of natural products shows wide-ranging biological activity and structural diversity. Some purine derivatives are also pharmacologically active [44–46]. Terpenylpurines are hybrid substances in which a terpene moiety (usually a diterpene moiety) is generally found as a substituent of position 7 of a 9-methyladeninium ion.

**Figure 1.**
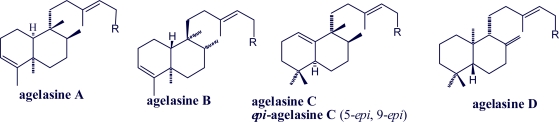

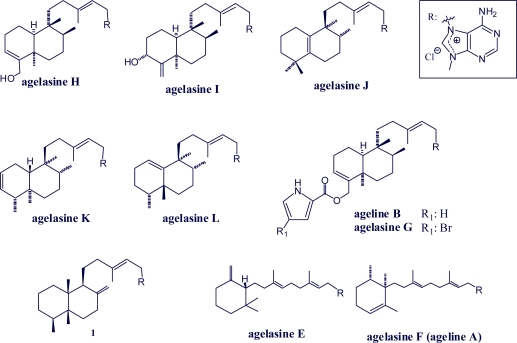
Structures of agelasines A–L.

The agelasines (Figure 1) are mono- or bi- cyclic diterpenoids having a 9-methyladeninium chromophore; they are quaternary adenine salts [47]. Ageline B and agelasine G have the general agelasine structure with a pyrrole hererocycle at diterpene moiety [37].

**Figure 2.**
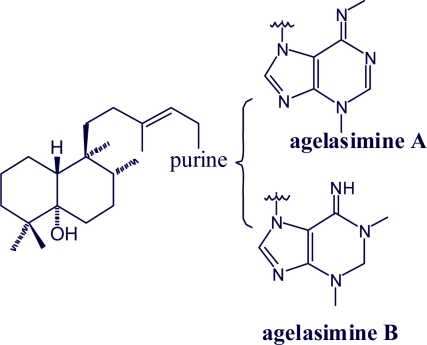
Structure of agelasimines A and B.

Agelasimine A and agelasimine B are adenine-related bicyclic diterpenoids and not quaternary adenine derivatives of a bicyclic diterpene [48,49] (Figure 2).

**Figure 3.**
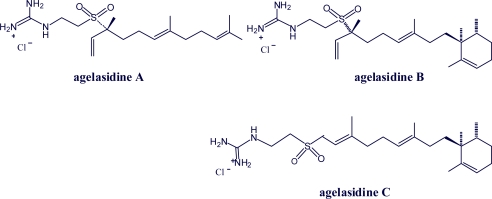
Structure of agelasidines A, B and C (guanidine derivatives, diterpene derivatives of hypotaurocyamine).

Agelasidines are not purine alkaloids. They are diterpene derivatives of hypotaurocyamine and have a guanidine unit. They may thus be considered as intermediate metabolites to agelasine F purine analogs [50] (Figure 3).

**Figure 4.**
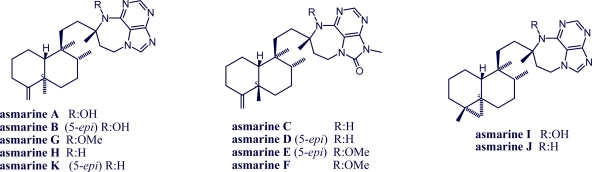
Structures of asmarines.

Asmarines are alkaloids with a unique tetrahydro[1,4]diazepino[1,2,3-*g*,*h*]purine (THDAP) structure. Asmarines are closest in structure to *Agelas* 9-methyladeninium-7-diterpenoids. However, they have a new heterocycle that includes a secondary hydroxylamine and they are not quaternary salts [38,51] (Figure 4).

**Figure 5.**
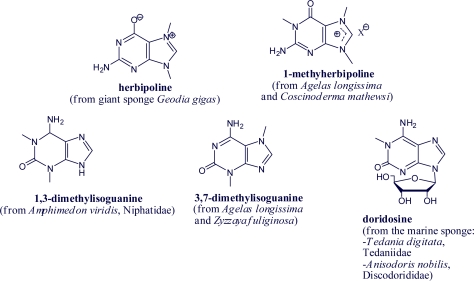
Methylpurines from marine organisms.

Other alkylpurines are the methyl-, dimethyl- or trimethylpurine derivatives isolated from different marine organisms [52–55]. Doridosine, for example, an adenosine analog, is a *N*-methylpurine riboside [56] (Figure 5).

## 3. Sources of Agelasines, Asmarines and Related Compounds

Agelasines have been isolated from Pacific *Agelas* sea sponges species and asmarines have been isolated from *Raspailia* marine sponge species. The genus *Agelas* (Porifera, Agelasidae) is an interesting and enigmatic genus of sponges from both the systematic and biogeographical points of view. There are 12 well-established species that are commonly found in tropical and subtropical shallow water environments, although their distribution is not homogeneous, as there are more *Agelas* species in the West Indies than in the whole of the Indopacific region combined. The genus *Raspailia* (Demospongiae, Poecilosclerida, Microcionina, Raspailiidae) is mainly distributed in the Red Sea and Indian Ocean. *Raspailia* sp. from the Indian Ocean are quite different from Red Sea species and appears zoologically closer to the Kenyan *Aulospangus involutos*.

The quaternary 9-methyladenine derivatives of the bicyclic diterpenes agelasine A-D and ageline B, and the monocyclic diterpenes agelasine E and F have been isolated from the Okinawan sea sponge *Agelas nakamurai* and from the Pacific sea sponge *Agelas* sp. [57–62]. Agelasine F was isolated from *Agelas sp*. collected in Baler, Aurora, Philippines [63]. Agelasine G was isolated from *Agelas sp.* collected off Konbu, Okinawa [37]. *Epi*-agelasine C was isolated from *Agelas mauritiana* [64]. Ageline B, agelasine F and agelasidine A were isolated from *Agelas sp* collected at Palau, Western Caroline Island [35]. Agelasine H and I were isolated from *Agelas mauritiana* collected at Yap Island in the Federated States of Micronesia [65]. Agelasine J, K and L [47] and agelasimine A and B [48,49] were isolated from the Solomon Islands orange marine sponge *Agelas mauritiana*. An agelasine **1** with an unusual thelepogane skeleton in the terpene moiety was isolated from the sponge *Agelas nakamurai* Hoshino collected in the Fly Islands, Papua, New Guinea [66]. Another new agelasine derivative has recently been isolated from the Caribbean sponge *Agelas clathrodes*[67], this compound has been called agelasidine J, even though it is described as a new 9-methyladeninium derivative. Agelasidine A was also isolated from *Agelas clathrodes* [50].

**Scheme 1.**
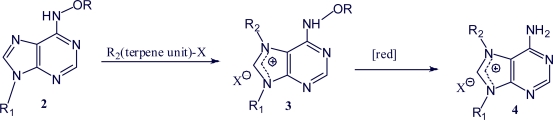
General synthetic route to agelasines [71].

Asmarines A-F were isolated from the marine sponge *Raspailia* sp. collected near Nakora Island, Dahlak Archipielago, Eritrea. [38,51,68]. Asmarines A, F, G and H were isolated from the Kenyan sponge *Raspailia* sp. [69]. Asmarines A, F, I, J and K were isolated from the Nosy Be Islands (Madagascar) sponge *Raspailia* sp. [70]. The wide range of biological activity makes these compounds attractive targets for synthesis. The general synthetic route to agelasines is represented in Scheme 1 (synthesis of agelasines **4** from **2**) [71].

Agelasines A-F have been synthesized. (−)-Agelasine A is also prepared using the enantiomerically homogeneous bicyclic iodide as a key intermediate for the total synthesis of the *cis*-clerodane diterpenoids [72]. *Trans*-clerodane (−)-agelasine B was prepared from an enantiomerically pure decalone. The key steps in the syntheses involve the stereoselective alkylation of the nitrile, the efficient coupling of the appropriate iodides to produce the clerodane skeleton, and electrochemical reduction to provide agelasine B [73]. Another route is adenine alkylation with the alkylbromide, obtained from methyl kolavenate by sequential reduction and bromination, to give, after reductive demethoxylation and ion-exchange chromatography, agelasine B [74,75]. Agelasine C has been prepared from *ent*-halimic acid [76].

**Scheme 2.**
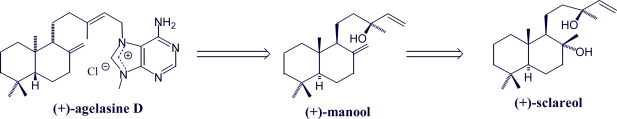
Retrosynthetic route to agelasine D [79].

The starting material for the terpenoid side chain on agelasine D is the readily available (+)-manool, and, at least formally, also the less expensive (−)-sclareol (Scheme 2) [77]. The synthesis was improved by Vik *et al*. [78]. Agelasine E was synthesized for the first time, together with analogs with various terpenoid side chains, by treatment of *N*_6_-methoxy-9-methyl-9*H*-purin-6-amine with allylic bromides, to give the desired 7,9-dialkylpurinium salts together with minor amounts of the *N*_6_-alkylated isomer. The *N*_6_-methoxy group was finally removed reductively [41,79].

**Scheme 3.**
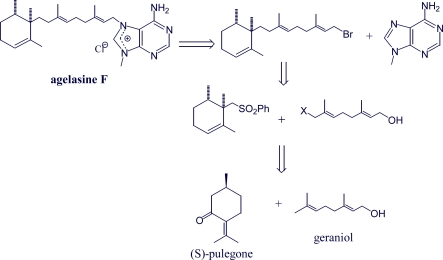
Retrosynthetic analysis of agelasine F [82].

Until 2009, only racemic agelasine F had been synthesized. That year Gundersen *et al.* reported [80] the first synthesis of *ent*-agelasine F, starting from (*R*)-pulegone. The synthesis is considerably more efficient than a previously reported route to *rac*-agelasine F (Scheme 3).

Simplified analogs of agelasines and agelasimines with a β-cyclocitral derived substituent have been prepared by Proszenyak *et al*. [81]. An illustrative summary of the synthesis strategies used to prepare agelasines can be found in reference [82]. The total synthesis of agelasimine A and B has been reported from (+)-*trans*-dihydrocarvone [48,49].

**Scheme 4.**
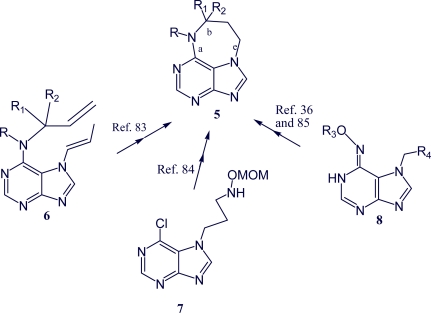
Synthetic routes to asmarines [83].

The heterotricyclic system of asmarines was prepared from suitably substituted purines (Scheme 4). Efficient construction of the clerodane decalin core of asmarines A and B has been achieved by an asymmetric Morita-Baylis-Hillman reaction/Lewis acid-promoted annulation strategy [86]. The tetrahydrodiazepinopurine ring skeleton **5** was prepared employing the ring-closing metathesis reaction on Boc-protected 6-allylamino-7-(propen-1-yl)purine **6** as the key step for the construction of the seven-membered ring. 7-(Propen-1-yl)purines were formed by a novel rearrangement of 7-allylpurines under basic conditions. Boc-protected *N*_6_,7-diallylpurine also participated in ring-closing metathesis reaction to give the eight-membered ring analog of the diazepinopurine [83]. Alternatively, tetrahydrodiazepinopurine ring skeleton **5** can be constructed by formation of bond “a” from compound **7** [84] and by formation of bond “b” from compound **8** [36,85]. Another methodology for the preparation of asmarine analogs was developed by Pappo *et al*. Three cyclization methods were applied to prepare the 9,9-disubstituted 10-hydroxy-tetrahydrodiazepino system: namely, aminomercurization, iodocyclization, and acid-catalyzed cyclization. The *O*-(3,4-dimethoxybenzyl group of the NOH functionality and cyanoethyl group of the *N*-9 atom were found to be the most suitable protecting groups [36].

In order to access a key component to complete the synthesis of asmarine B, Rodgen *et al.* [87] developed a methodology involving the allylboration of imines followed by subsequent oxidation to form the desired hydroxylamine. Nevertheless, the total synthesis of asmarine B has still not been published, although other synthetic strategies towards key asmarine intermediates have [88,89].

## 4. Biological Activity of Marine Terpenyl-Purine Alkaloids

### 4.1. Antifouling activity

There is an increasing interest in exploring the antifouling potential of natural products because there is a clear need to develop new non-toxic or environmentally benign antifouling alternatives that will be efficient against the most severe fouling organisms such as barnacles, blue mussels, bryozoans and algae. Many sponges have been shown to synthesize toxic metabolites to prevent predation, and hence other organisms frequently attach sponges to themselves for their protection [90].

Agelasine D, together with two of its analogs, displays a strong inhibitory effect on the settlement of *Balanus improvisus* cypris larvae (EC_50_ 0.11–0.30 μM). None of these three compounds affected larval mortality. When after 24 h exposure to the compounds the cyprids were transferred to fresh seawater, the settlement frequency was completely recovered in comparison with the controls. These properties make agelasine D and analogs highly attractive candidates as antifouling agents in future marine coatings [43]. The antifouling activity of *epi*-agelasine C against *Ulva* spores is not as high as that of CuSO_4_ (positive control); however, *epi*-agelasine C shows lethal activity against *Ulva* fronds at 50 ppm. Antimicroalgal activity against *Oscillatoria amphibian* (Cyanophyceae), *Skeletonema costat*um (Diatomophyceae), *Brachiomonas submaria* (Chlorophyceae) and *Prorocentrum micans* (Dinophyceae) has been observed at 1.0–2.5 ppm, so this compound seems to be useful as a measure to counter red tide [64].

### 4.2. Antituberculosis activity

Tuberculosis is still a major health problem worldwide. Although the treatment regimens currently available can cure almost all tuberculosis drug-susceptible cases, problems such as the length of treatment, the need for multidrug therapy, the emergence of drug resistance, HIV co-infection, and persistent *Mycobacterium tuberculosis* bacilli, highlight the need for new anti-tuberculosis drugs. New anti-tubercular drug regimens are clearly needed to reduce the time required for a lasting cure and to treat the expanding problem of drug- and multidrug-resistant (MDR) *Mycobacterium tuberculosis* strains [91,92]. The strategies to search for new antituberculosis drugs involve screening libraries of small molecules and natural products or the previous identification of targets crucial to the microorganism, followed by the subsequent design of new molecules. Antituberculosis compounds from natural sources have enormous potential for the development of new drugs that have shown not only antimicrobial activity *per se* but also inhibition of the mechanism of resistance (e.g., efflux pumps) or modulation of the immune response (e.g., macrophage stimulation) [93,94].

*In vitro*, agelasine F inhibits some drug-resistant strains of *Mycobacterium tuberculosis* and inhibits the growth of tuberculosis H_37_Rv at concentrations as low as 3.13 μg/mL. The metabolite is also equally potent against a range of single drug-resistant strains including, isoniazid, rifampicin, ethambutol and ethionamid. Activity against *M. tuberculosis* residing within macrophages requires concentrations of 13–22 μg/mL, which are below the IC_50_ for Vero cells (34 μg/mL) [63,82]. The weak bactericidal activity of agelasine F, indicated by a higher value for EC_99_, together with the moderate toxicity to Vero cells, disqualifies agelasine F as a drug *per se*. However, it might serve as an interesting lead for the design and synthesis of a more active molecule [63].

Only modest antimycobacterial activity has been found for agelasine E [34] and agelasine analogs with free NH_2_ in the position 6 of the purine, but several agelasine analogs still carrying the MeO-directing group at *N*-6 are highly potent inhibitors against *M. tuberculosis.* A relatively long *N*-7 side chain is required for significant activity. *M. tuberculosis* has an extremely thick and waxy cell wall, which is an effective barrier for many chemicals. Accordingly, effective drugs should have a reasonable degree of lipophilicity in order to penetrate that wall. This may explain why more polar compounds are generally less efficient than less polar ones. However, in some *N*-6-alkylated agelasine isomers, formed as by-products in the alkylating step, significant activities have been found when the terpenoids side chain is relatively large. Furthermore, while the *N*-6-methoxy-9-methyladenine is essentially inactive, the simple 9-methyladenine exhibits an MIC against *M. tuberculosis* at 6.25 μg/mL of 94% (positive control, rifampicin) [41]. Reasonable antimycobacterial activity has also been reported for some simple 9-benzyladenines [95]. 7-Alkyl, 7-benzyl and 7-geranyl purinamine derivatives are more or less inactive against the bacteria examined. In contrast, (2*E*,6*E*)-farnesyl and isomeric (2*E*,6*Z*)-farnesyl derivatives exhibit a strong inhibitory activity against *Mycobacterium tuberculosis* [34].

### 4.3. Antimicrobial activity

Infectious diseases caused by bacteria, fungi, viruses and parasites are still a major threat to public health despite the tremendous progress in human medicine. Natural products, either as pure compounds or as standardized extracts, provide unlimited opportunities for new anti-infective drug leads because of the unmatched availability of their chemical diversity [96].

Some agelasine analogs show antibacterial activities. Agelasine D and close analogs display a broad spectrum of antibacterial activities, including effects on *M. tuberculosis*, Gram-positive and Gram-negative bacteria (both aerobes and anaerobes). (2*E*,6*E*)-farnesyl and isomeric (2*E*,6*Z*)-farnesyl derivatives, on the other hand, exhibit a profound inhibitory activity against *Staphylococcus aureus*. These compounds are also active against *Bacteroides fragilis* and *Bacteroides thetaiotaomicron* (anaerobe). The geometry of the terpenoid side chain appears to have no significant influence on antibacterial activity. Geranylgeranyl purine is also active against *Streptococus pyrogenes* and *Enterobacter facealis*, but not against *Pseudomonas aeruginosa*, in the concentration range examined. Reduced inhibitory activity against bacteria has been found for phytyl derivatives, indicating that the unsaturation in the side chain is important for the antibacterial activity [34,35].

Several samples of *Agelas* sp. containing agelasine F, agelasidine A and ageline B, show antimicrobial activity against Gram-positive bacteria *S. aureus* and two fungi: *Candida albicans* and *C. utilis* [63]. Pure samples of ageline B and angelasine F inhibit the growth of *S. aureus*, *B. subtilis* and *C. albicans*. Agelasine F and agelasidine A are lethal to goldfish, *Carassius auratus* (ichthyotoxicity) [65].

*Ent*-agelasine F exhibits antimicrobial activity: MIC *Staphylococcus aureus* 2 μg/mL, and MIC *Escherichia coli* 16 μg/mL [80]. *Epi*-agelasine C does not exhibit antibacterial activity against *Staphylococcus aureus, Vibrio costicola, Escherichia coli* or *Bacillus subtilis* [64].

Agelasine A and H are inactive against *P. aeuroginosa, S. aureus*, *Aspergillus niger* and *Saccharomuces cerevisiae* at concentrations of 200, 100, 10 and 1 μg/mL. Agelasine B and ageline B inhibit *S. cerevisiae* at 10 μg/mL. Agelasine F inhibits *A. niger* at 100 μg/mL and *S. cerevisiae* at 10 μg/mL. Agelasine I **7** inhibits *S. cerevisiae* at 200 μg/mL[65]. Agelasimine analogs are strong inhibitors of *S. aureus* and *E. coli* [34].

### 4.4. Antiprotozoal activity

Agelasine D, other agelasine analogs and related structures have been screened for their inhibitory activity against *Plasmodium falciparum, Leishmania infantum, Trypanosoma brucei and Tripanosoma cruzi*, as well as for toxicity against MCR-5 fibroblast cells, in order to assess the selectivity of their action [40]. A higher activity was found for agelasine D (IC_50_ 0.63 μM) against *P. falciparum* than agelasine J (IC_50_ 6.6 μM), agelasine K (IC_50_ 8.3 μM) and L (IC_50_ 18 μM). The selectivity index of agelasine D for antimalarial action [SI: IC_50_ (MCR-5 fibroblast)/IC_50_ (*Plasmodium falciparum*)] was 23 and it also displays a significant inhibitory action against the other parasites examined. According to criteria set up by the WHO Special Programme for Research & Training in Tropical Diseases (TDR), in that study two agelasine analogs were identified as hits for leishmaniasis and for Chagas’ disease [97]. Agelasine J, K and L display *in vitro* antimalarial activity against *Plasmodium falciparum* [47].

### 4.5. Cytotoxic activity

Some agelasine and agelasimine analogs exert a strong cytotoxic activity against several cancer cell lines (MIC 0.1 μM for the most potent compound), including a drug-resistant renal cell line [80].

Agelasine G shows cytotoxic activity against lymphoma L1210 cells *in vitro*, with an IC_50_ value of 3.1 μg/mL [37]. The agelasine analog (2′*E*,6*E*,10′*E*)-6-methoxyamino-9-methyl-7-(3,7,11,15-tetra-methyl-2,6,10,14-hexadecatetraenyl)-7*H*-purinium, exhibits a powerful activity against cancer cell lines. This agelasine analog is also a potent inhibitor of ACHN (renal adenocarcinoma cells) growth and is more effective against the primary multidrug-resistant (MDR) cell line ACHN than any of the anticancer drugs used as positive control (doxorubicin, cisplatin and paclitaxel) [34].

Asmarines A and B have cytotoxic activity against cell cultures of P-388 murine leukemia, HT-29 human colon carcinoma, and MEL-28 human melanoma cells. Asmarine B (IC_50_ 0.12–0.24 μM) is more active than asmarine A (IC_50_ 1.18 μM) and shows higher activity against human lung and human colon carcinoma [51].

Asmarines A and B and some synthetic analogs have been tested for cytotoxic activity against DU-145 prostate, IGROV-ET ovarian, A-549 NSCL, PANC1 pancreas and LOVO colon cancer cell lines. Synthetic compounds were all found to be at least one order of magnitude less active than asmarine B (GI_50_ 0.04–0.5 μg/mL). The cyclization modes for 9-mono and 9,9-disubstituted tetra-hydro[1,4]diazepino[1,2,3-*g*,*h*]purine structure can form the basis of SAR studies of asmarine analogs, and provide a route for the total synthesis of asmarine A starting from the appropriate diterpene and purine [36]. The biological target of asmarine A and B is not known [86].

### 4.6. Inhibitory effect on the enzymatic reaction of Na^+^, K^+^-ATPase

It is well established that Na^+^,K^+^-ATPase hydrolyzes ATP to provide the energy for the active transport of Na^+^ and K^+^ across the cell membrane. This enzyme acts as the electrogenic Na^+^, K^+^ pump and contributes to the membrane excitability of several cells. Inhibition of Na^+^,K^+^-ATPase has physiologically important roles, such as the cardiotonic effect [98].

The Na^+^,K^+^-ATPase from brain or kidney and sarcoplasmic reticulum Ca^2+^-ATPase were potently inhibited by agelasidine C and agelasine B and exert a less potent inhibition on heart Na^+^,K^+^-ATPase. The ionized moiety of agelasidine C and the long nonpolar side chains in agelasine B play important roles in their inhibitory action. The inhibition of Na^+^,K^+^-ATPase by agelasidine C or agelasine B is almost reversed by diluting with inhibitor-free solution [42]. Agelasin B and some analogs show inhibitory activity against Na^+^,K^+^-ATPase reactions at 10^−4^ M [99].

Other activities of terpenylpurines, such as the inhibition of adenosine transfer into rabbit erythrocytes, their Ca^2+^-channel antagonistic action and an α_1_ adrenergic blockade, have been reported [65].

Moreover, the biological properties of some methylpurines are also very interesting. *In vivo* doridosine causes hypotension, a reduction in heart rate, muscle relaxation and anti-inflammatory effects through adenosine A_1_ and A_2_ receptors [56,100]. The affinity prediction on A_1_ adenosine receptor agonists has been determined by the chemometric approach [101]. 1-Methylherbipoline is a serine protease inhibitor [52].

## 5. Summary

Some natural products have a profound impact on human health. The biosynthetic engine of Nature produces innumerable secondary metabolites with distinct biological properties that make them valuable as health products or as structural templates for drug discovery.

Marine sponges of the genera *Agela* and *Raspailia* have been demonstrated to be a rich source of bioactive alkaloids. Many terpenylpurine alkaloids isolated from those genera are of considerable interest with regard to their biological activities. Furthermore, some of them have exhibited promising activities in future marine coatings as antifouling alternatives against the most severe fouling organisms, one of the most serious problems of marine technology.

Agelasines, asmarines and related structures display cytotoxicity and antiprotozoal, antimicrobial, antituberculosis activity, among others. Identification of the hits as well as other SAR studies will be valuable for the design of more potent and selective potential drugs against several diseases.
